# Electrochemical Impedance Spectroscopy Investigation on the Clinical Lifetime of ProTaper Rotary File System

**DOI:** 10.1155/2014/754189

**Published:** 2014-01-29

**Authors:** Virgil Penta, Cristian Pirvu, Ioana Demetrescu

**Affiliations:** Faculty of Applied Chemistry and Materials Science, Polytechnic University of Bucharest, Polizu 1-7, 011061 Bucharest, Romania

## Abstract

The main objective of the current paper is to show that electrochemical impedance spectroscopy (EIS) could be a method for evaluating and predicting of ProTaper rotary file system clinical lifespan. This particular aspect of everyday use of the endodontic files is of great importance in each dental practice and has profound clinical implications. The method used for quantification resides in the electrochemical impedance spectroscopy theory and has in its main focus the characteristics of the surface titanium oxide layer. This electrochemical technique has been adapted successfully to identify the quality of the Ni-Ti files oxide layer. The modification of this protective layer induces changes in corrosion behavior of the alloy modifying the impedance value of the file. In order to assess the method, 14 ProTaper sets utilized on different patients in a dental clinic have been submitted for testing using EIS. The information obtained in regard to the surface oxide layer has offered an indication of use and proves that the said layer evolves with each clinical application. The novelty of this research is related to an electrochemical technique successfully adapted for Ni-Ti file investigation and correlation with surface and clinical aspects.

## 1. Introduction

Modern endodontics require a predictable result in an area where such a result is difficult to obtain in classical conditions. With recent advances in electronics and material's science, the endodontic micromotor and flexible Ni-Ti files have emerged. An initial investigation on the bending and torsional properties of nitinol (Ni-Ti) root canal files has been introduced in the pioneering article by Walia et al. [1]. This article noted the advantage of the low elastic modulus Ni-Ti alloy for negotiating curved root canals, compared to the stainless steel alloys in predominant use at the time. The nickel-titanium endodontic instruments reported by Walia et al. were intended for hand usage, and the rotary instruments employed with slow-speed dental handpieces followed in the 1990s. Currently, there are many types of rotary file systems on the market but this paper focuses on the ProTaper. This system is comprised of six files that are used in sequence to enlarge and reshape the endodontic root canal conforming it to Schilder's principles [2]. The first three are named shaping Sx, S1, and S2 and the second three are named finishing F1, F2, and F3; these files are the basic Maillefer Dentsply kit presented by West [3]. They are a leap forward in root shaping and enlargement. They offer great results and are very time efficient in root preparation as shown by Tu et al. [4]. One of the main problems with the ProTaper files is the frequent breakage, a fact presented by many different studies, Fife et al., Inan et al., and Lopes et al. [5–7]. The actual issue is not necessarily the remainder of the file fragment within the root canal but the pathology resulting from incomplete canal therapy as shown by Nair [8]. This character of Ni-Ti files makes it a matter for dental materials science to improve and perfect a technique by which the doctor could easily quantify their clinical lifetime, Plotino et al. [9]. The cause for file failure is complex and resides in many areas such as alloy fatigue, improper use, corrosion due to lavage substances, and operator sensitivity as presented by many studies, West, Lopes et al., Peters et al., and Cheung and Darvell [3, 7, 10, 11].

The electrochemical behavior of Ti alloys in the oral cavity environment has been widely investigated in the last decade taking into account alloy and environment composition [12, 13]. Various compositions of binary and tertiary alloys have been subject of such research taking into account their applications as dental materials due to mechanical properties as well [14]. As binary alloy, Ti-Ni has attracted much attention of researchers being a shape memory alloy with good corrosion and abrasion resistance used in orthodontic arch wires and rotary endodontic instruments.

This paper compares 13 ProTaper clinically utilized sets in order to assess the use of EIS in determining the instruments clinical lifetime and possible risk of fracture. The files are made of a Ni-Ti memory shape alloy as shown by Alapati et al. and Baek et al. [15, 16]. The alloy has great elasticity but in time and due to stress, it wears out and structural defects under the form of cracks appear; the cracks are points of low resistance as pointed out by different studies such as Sattapan et al. [17], Luebke et al. [18], and Gambarini [19]. Furthermore, the liquid used for root lavage is sodium hypochlorite; it infiltrates the cracks and corrodes deeper into the file, a fact presented by Lopes et al. and by Peters et al. [7, 10]. The result is file breakage during enlargement. When new, the ProTaper files are covered with a homogenous layer of oxide that protects the alloy. This oxide layer is essential to the resistance of the Ni-Ti file because it acts like a barrier between the file and the outside environment. The advantage of such a layer is fast regeneration when exposed to oxygen in breathable air. During instrumentation, the oxide layer is mechanically removed and the alloy becomes exposed to corrosive substances like sodium hypochlorite as shown by different studies (Peters et al., Cheung and Darvell [10, 11]). If the file is placed into a liquid disinfecting solution before it can regain its oxide coating then the file can corrode even faster especially in the areas of oxide layer lack.

Electrochemical impedance spectroscopy method, which has seen tremendous increase in popularity in recent years, is a useful tool to evaluate the electrochemical stability of oxide surface films and to identify/evaluate the surface modification for Ni-Ti alloys [20].

The EIS investigation is based on the system response to the application of a periodic small amplitude a/c signal. The measurements are carried out at different a/c frequencies and the system response contains information about the interface, its structure, and reactions taking place there [21].

In our case, this procedure reveals the state of the titanium-oxide layer in direct connection to the usage of the instrument. A new instrument will be quite capacitive showing a homogenous and uniform oxide layer as opposed to a used instrument that has a more resistive character because of modification in oxide layer coating. This approach has been introduced in an effort to extend EIS use to the dental field, to offer a new perspective to clinicians on eclectic oral interactions as shown in our previous papers [22, 23]. The principle is that electrons will only circulate on the superficial part of the Ni-Ti alloy and diffusion is seen through the oxide layer revealing its characteristics. Although there has been a very interesting proposition of treating the fatigue of the files using Griffith's law, it is the authors' opinion that such a model is only a partially valid one and the application of the said law or of Irwin's modification cannot describe the complexity of the clinical situation as shown by Cheung and Darvell [11].

## 2. Materials and Method

### 2.1. Files

Thirteen ProTaper Dentsply/Maillefer sets used clinically on patients and a new reference set, in total a number of 84 files, were tested [3]. The ProTaper technique is a crown-down root preparation principle in which each instrument has changing percentage tapers over the lengths of its cuttings blades [3]. The files were all collected from the same dental clinic and from the same practitioner in order to minimize the differences of operator sensitivity and technique, Plotino et al. [9]. By gathering all the files from the same place, we believe that we can increase the probability that they have been used very much in the same way with the same technique and by the same trained hand. Previous articles show that a number of about 8–12 root canals can be enlarged with the same file set, Fife et al. and Inan et al. [5, 6]. These root canals must be quite straight with a maximum curvature of 10 degrees in order to maintain the 12-canal quantity; otherwise the number of uses must decrease, Fife et al. [5]. Unfortunately, this is quite a subjective matter and it is entirely up to the trained specialist to decide when to discard each set.

### 2.2. EIS Testing

Electrochemical impedance spectroscopy (EIS) measurements were conducted with Autolab PGSTAT 302 N in a logarithmic distribution range between 100 kHz and 10 mHz. The EIS fitting was performed using NOVA 1.8 software. The electrochemical setup consisted of a three-electrode configuration with a counter platinum electrode, an Ag/AgCl electrode as a reference, and the file itself as the working electrode. Each file was submerged to the same reference point 1 mm under the file haft in an electrochemical cell. The results may be represented graphically using two types of plots: complex plane Nyquist plots (*Z*′′ versus *Z*′, i.e., the imaginary versus the real components of the impedance, plotted for various frequencies) and Bode plots (log⁡⁡|*Z*| (magnitude) and phase-angle, *ϕ*, versus log *ω* (angular frequency) [21].

After testing, all files were compared with the same type in each set and a number of conclusions were formulated. The surface observations of each file were done with Carl-Zeiss optical microscope up to 50x magnification.

### 2.3. Set Usage Registered and Described Using EIS

After comparing each type of files, we have compared each set wear to the clinical use registered by the practitioner. Each file is dipped in Glide solution before the actual use and each use is accompanied by copious irrigation with sodium hypochlorite 2.5%. The clinician always uses the Sx files but not before the confirmation of the Glide path up to Headstroem 15 file. Standard rubber dam isolation is routinely done for every case. Set numbering in this paper is in direct connection to number of clinical uses as follows.

The impedance values described for each set represent a mean of the impedance values of each file of the set.

The reference value used for the discussion of file set lifespan and service life was that of 48.34 Ohms value registered by the reference unused ProTaper set.

Set 1 is an example of minimal file wear. It has been used to enlarge a maxillary front incisor 1.1 with a morphology described by Kerekes and Tronstad [24]. It has been used in our study to grossly quantify the modification of the oxide layer after a single use in a straight root canal. Its average impedance value was 54.21 Ohms and the total number of canals enlarged was 1 (Figures 8 and 9).

Set 2 has been used in the instrumentation of two front incisors 2.2 and 2.1. The set was discarded for research purposes only and does not show any objective wear either using direct observation or the microscope. Further use world strongly be recommended. Its average impedance value was 59.24 Ohms and the total number of enlarged canals was 2 (Figure 8).

Set 3 was used in the instrumentation of 2 maxillary canine teeth and a maxillary premolar tooth, 1.3, 2.3, and 1.5. The microscope image of the files shows no surface modification or defect. Its average impedance value was 58.73 Ohms and the total number of enlarged canals was four (Figures 8 and 9).

Set 4 was used for the enlargement of 2 maxillary molars 1.7 and 1.8. Tooth 1.8 had a two-canal morphology with an extremely distally curved single root, Kerekes and Tronstad [25]. Also one of the canals had a broken tip of probably a Kerr file that could not be removed. The set shows accentuated wear of the S1, S2 and F1, F2 files (Figure 3(a)). The average impedance value was 118.71, a very high value although the total number of enlarged canals was 5 (Figures 8 and 9).

Set 5 has been used in the instrumentation of two mandibular molars 4.6 and 3.6 in the same patient. So in total, a number of 6 canals have been instrumented. The microscope showed visible defects correlating to high impedance values as shown regarding the S1 file (Figure 3(b)). The average impedance value for this set was 64.14 Ohms and the total number of canals was 6 (Figure 8).

Set 6 has been used for the instrumentation of three maxillary premolars 1.4 and 1.5 and a special anatomy of a 2.5 premolar with 2 very narrow and convergent canals, Kerekes and Tronstad [25]. The strange anatomy of the maxillary premolar could account for the excessive wear of S1 and S2 (Figure 3(c)). The average impedance value for this set was 89.30 and the total number of enlarged canals was 6 (Figure 7).

Set 7 was used in the instrumentation of 3 upper maxillary molars in a young patient aged 23 years old. The set shows notable modification upon direct observation viewed by the microscope (Figure 3(d)). The average impedance value for this set was 67.55 Ohms and the total number of enlarged canals was 9 (Figure 8).

Set 8 has been used for 3 molars in two different patients. The teeth instrumented were 1.6, 4.7, and 2.6. The set shows high EIS values and minimal defects were viewed with the optical microscope. The average impedance value for this set was 76.85 Ohms and the total number of enlarged canals was 9 (Figure 8).

Set 9 was used for the instrumentation of 3 mandibular molars in two different patients 4.6, 4.7, and 3.6. The roots were normally conformed. The average impedance value was 77.26 Ohms and the total number of enlarged canals was 9 (Figure 8).

Sets 11, 12, and 10 have been heavily used for a number of 12 canals each. Sets 12 and 11 have been used in the instrumentation of 4 mandibular molars each. Set 12 enlarged 4.6, 3.6, 4.7, and 3.8 with 2-root-3-canal morphology and high curvature. Set 11 enlarged 3.8, 4.8, 3.6, and 3.7 teeth. Tooth 4.8 presented a convergent shape of its mesial and distal canals, Kerekes and Tronstad [26]. The set number 10 was used in the enlargement of five maxillary teeth; four maxillary premolars: three 1.4, one 2.4 and a central incisor 1.1. The average impedance values for these sets were, for set 10: 87.79 Ohms, for set 11: 88.04 Ohms, and for set 12: 90.92 Ohms (Figure 7). Set number 10 enlarged a number of 11 root canals and sets 11 and 12 enlarged a number of 12 dental root canals. All the sets described above show high impedance values and surface defects when viewed with the microscope especially set number 12 and should be readily discarded (Figures 3(e), 8, and 9).

Set 13 has been used for 5 molars in two different patients enlarging a number of 15 dental canals. The molars were 3.6, 1.6, and 1.6 and mandibular right molars 4.6 and 4.7. Files F1 and F2 were used in each case but file F3 has only been used in the instrumentation of the distal roots of each molar so this accounts for the lower wear. The average impedance value for this set was 126.67 and the total number of enlarged canals was 15 (Figure 8). Surface defects were viewed with the optical microscope (Figure 3(a)).

After the consideration of all sets, a few conclusions can be formulated. The first would be that the modification of impedance values occurs not with the frequency of clinical use but with the difficulty of each case in part. It is apparent that set 4 has less uses than other sets but it presents a pattern of wear specific to a higher frequency of clinical use. So, it is very important to show that the EIS data correctly quantifies modification of oxide layer relative to usage and wear.

Although the most worn files showed many surface defects in the form of pitting corrosion, scratches or even missing pieces of alloy, the F1 in Set 4 presented a crack in the file structure posing a breakage risk for future clinical use (Figure 3).

## 3. Results and Discussion

**Figure 1:**
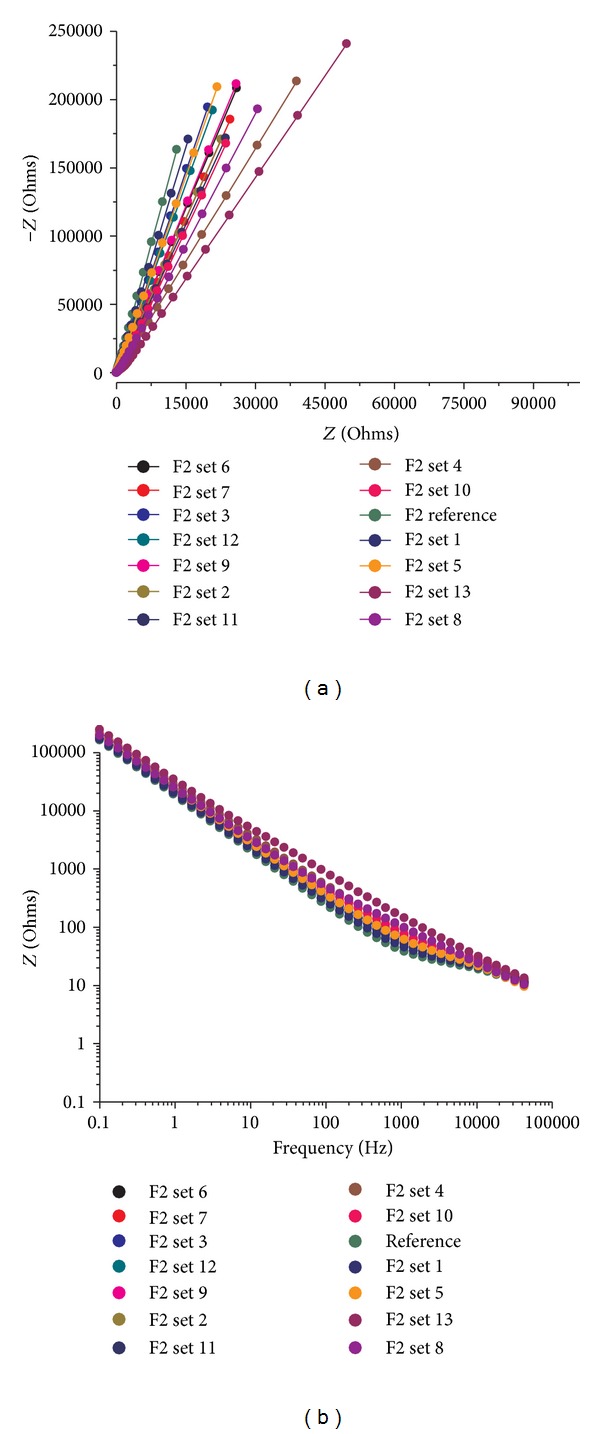
Nyquist and Bode diagrams of S2 files from all sets showing progressive wear starting from reference on (a) and most worn file on (b).

**Figure 2:**
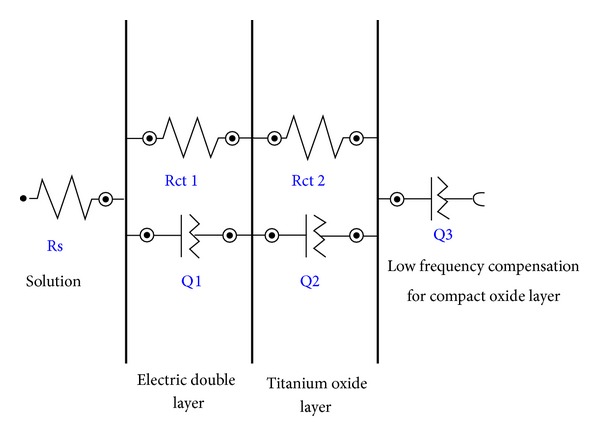
Equivalent circuit for EIS fitting.

The results of file testing showed a variation in impedance values by comparison to the unused set. The Nyquist diagram versus Bode plot in Figure 1 shows how the electrochemical behavior of the titanium-oxide layer on the files changes with several uses. The Ti-oxide layer changes with multiple uses, so, we measured a new unused file as a reference and then we measured a file with many clinical uses, 15 enlarged canals, file F1 set 13. The change was drastic and very easy to spot on the EIS plots and in correlation to the fitting circuits (Figure 2).

**Figure 3:**
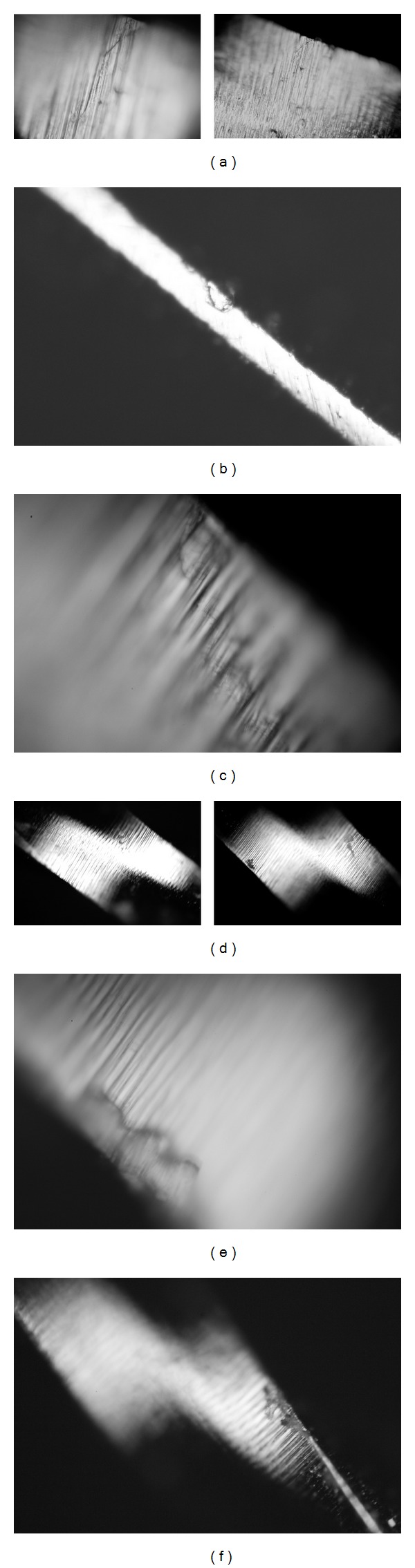
Optical microscope images: (a) F1 set 4, (b) S1 set 5, (c) S2 set 6, (d) F1 set 7, (e) S2 set 12, and (f) F1 set 13.

In order to confirm that impedance value variation of the oxide layer resounds in alloy defects, we analyzed the used F1 from set 13 file using the Carl-Zeiss optic microscope and we found cracks in its structure (Figure 3(f)). We repeated this algorithm with all the file classes and we found that there is a very clear connection between clinical use and oxide layer modification. Our limits were placed having as a reference a new set and as a limit to usage the clinically overworked files with very high resistances regardless of set number that presented structural defects under the microscope. The Nyquist values showed a change in electrical impedance toward an increase. The complexity of evaluating the files with this kind of plots was too great so we preferred to use Nyquist only for a qualitative interpretation of oxide layer change and explanation of surface phenomena. The quantification of resistance variation was done using Bode modulus plots at a frequency around 10000 Hz simply displaying the relation between frequency used and resistance modification.

### 3.1. Fitting Circuit

The circuit in Figure 2 is an example of circuit used for EIS data fitting. According to the impedance spectra in the metal/oxide/electrolyte configuration, the equivalent circuit, shown in Figure 2, represents the impedance behavior of the oxide films. In this circuit, Rs represents the aqueous solution resistance. The two observed charge-transfer flattened semicircles correspond to two RC parallel combinations and are due to the ionic charge transfer resistance, Rct1 in parallel to the first constant phase element (Q1) and the Rct2 in parallel to the second constant phase element (Q2).

A constant phase element (CPE) is generally considered a component that models the behavior of a double layer, that is, an imperfect capacitor (pseudocapacitor). In global measurements of an irregular surface, the CPE behaviour can be attributed to either different surface or normal time-constant distributions. Thus, normal distributions of time-constants can be expected in systems such as irregular oxide surfaces as shown by Jorcin et al. [27].

The equivalent electric circuit contains a supplementary constant phase element (Q3) for lower frequency corresponding to the compact oxide coating and indicating the pseudocapacitive behavior of these films.

The circuit corresponds to the electrochemical events at the interface of file and solution and describes diffusion through the oxide layer. Based on the values of each circuit component, we can compare different files and better understand their qualities and wear pattern.

### 3.2. Quantification of Oxide Layer Change per One Use

**Figure 4:**
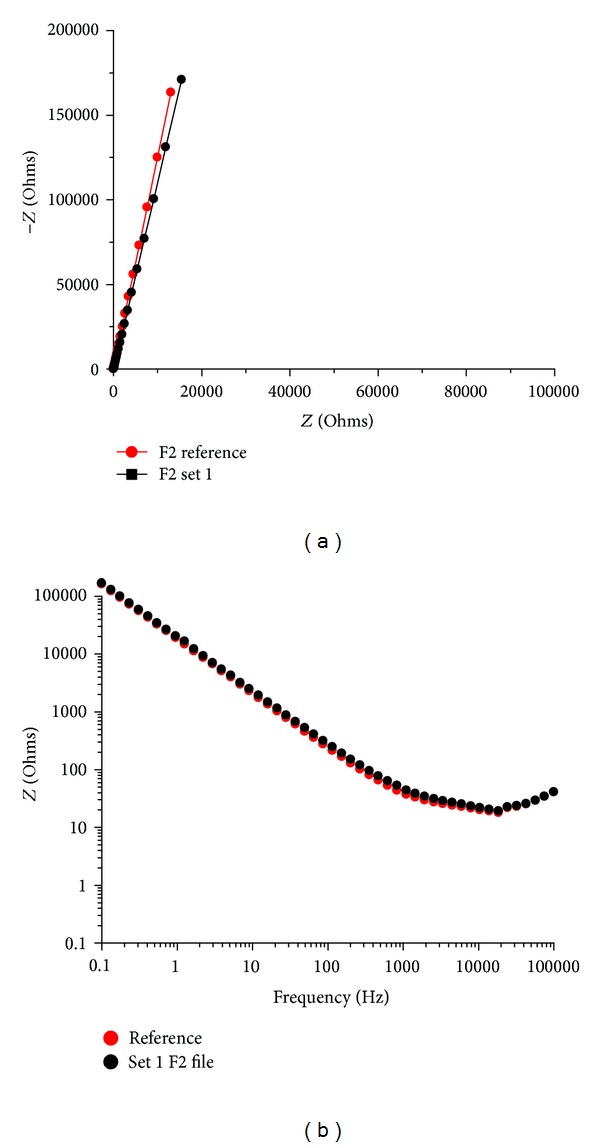
Comparison between reference set and set 1 F2 file with only one clinical use on a singular root incisor in order to quantify oxide layer change per one use.

We wanted to go even further and try to grossly quantify oxide layer modification per one use in an ideal straight canal. For this purpose, we chose a front maxillary tooth 1.1 and enlarged it to the F3 file. Then, we measured the files using EIS and computed the difference in resistance to the new unused set (Figure 4).

**Figure 5:**
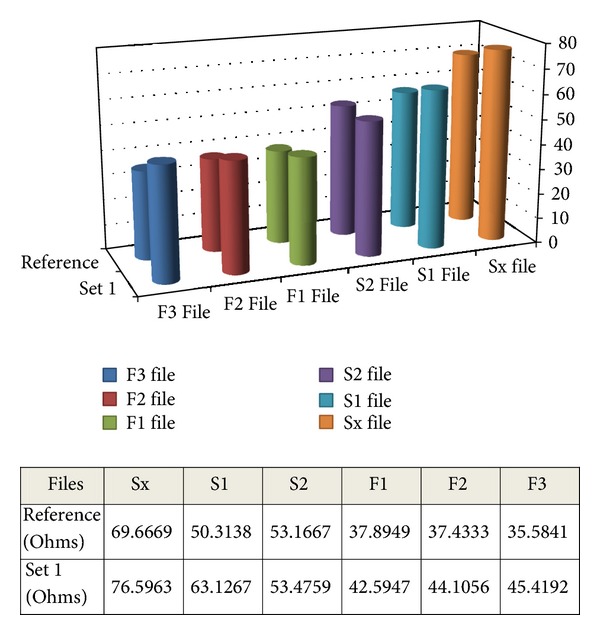
Comparison between reference set and set 1 with only one clinical use on a singular root incisor in order to quantify oxide layer modification per one use.

**Figure 6:**
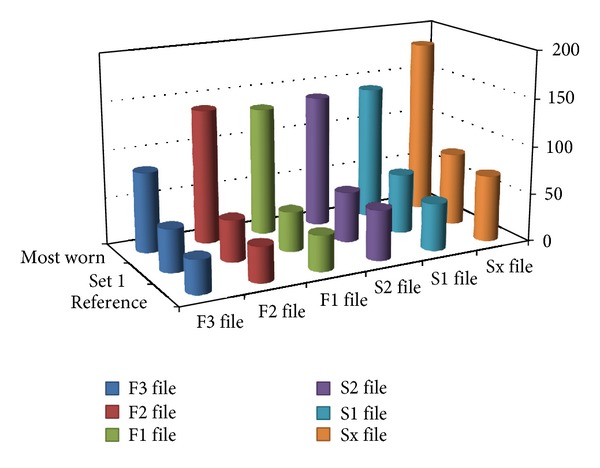
Comparison between reference set and set 1 with one use and the highest impedance value files in the whole lot.

Figures 5 and 6 also summarize the measurements described above and display a very interesting surface quality of the files. Although the surface modification for one use is important, these files can theoretically resist a great number of wear cycles in straight canals. So in theory, we could use these files for a much greater number of canals than we actually do. The difference in real-life use is that corrosion together with mechanical strain wears the files at a much faster rate, Viana et al. [28].

### 3.3. General File Description Using EIS

#### 3.3.1. Shaping Files

**Figure 7:**
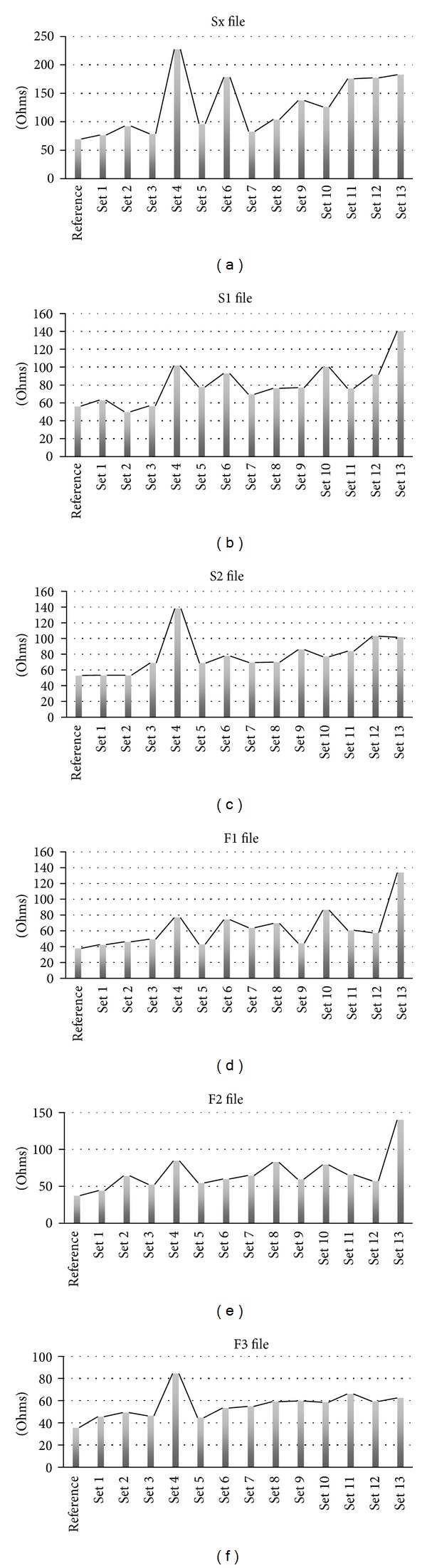
Figure showing pattern of impedance value modification for each of the six files in turn.

Sx—This is a file used for the initial root canal access enlargement. A brushing action is indicated while using this file as presented by many authors, Luebke et al., Gambarini, and Ruddle [18, 19, 29]. In Figure 7, we can observe the relation of all the files to the reference Sx and we can quantify the oxide layer of each file to the reference one. By measuring the reference Sx file, we found a value of approximately 69.6 Ohms. The highest resistance is set number 4, 227.237 Ohms, roughly three times the initial value. This set contains the highest impedance value of an Sx file. As a general quality of Sx files, they tend to have a higher increase in impedance values even with a reduced number of uses. This characteristic could be due to their unique shape (Figure 7).

S1—Shaping 1 is the first of the shaping files in the ProTaper set; it is very thin and flexible. It enlarges the upper parts of the canal toward the middle of the canal. Its tip is very thin and flexible and should be able to spin freely into the canal, a fact presented by Luebke et al. [18]. The reference impedance value is approximately 56.3 Ohms. Set 13 file registers the highest value of 140.3 Ohms representing the most worn S1 (Figure 7).

S2—The S2 files enlarge the middle and lower part of the canal but do not interfere in the apical stop area. Being less flexible, it suffers more from the effects of bending into curved canals. The reference value for these files registered at 53.16 Ohms and the highest impedance value obtained was in set number four, 138.1 Ohms (Figure 7).

**Figure 8:**
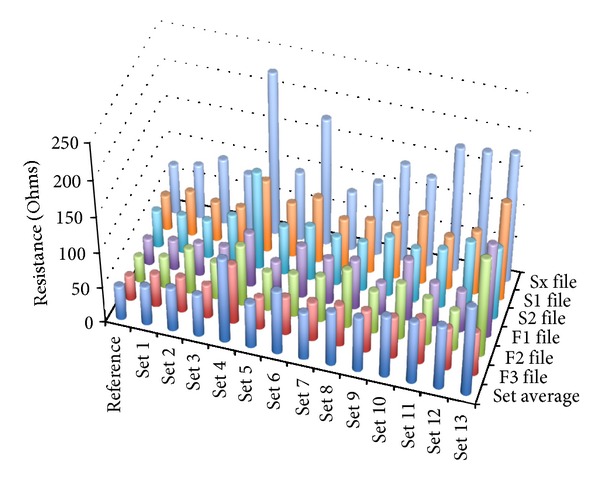
Figure showing wear of files and sets compared to reference files. The height of each column shows the wear value of each file based on ohmic resistance modification.

**Figure 9:**
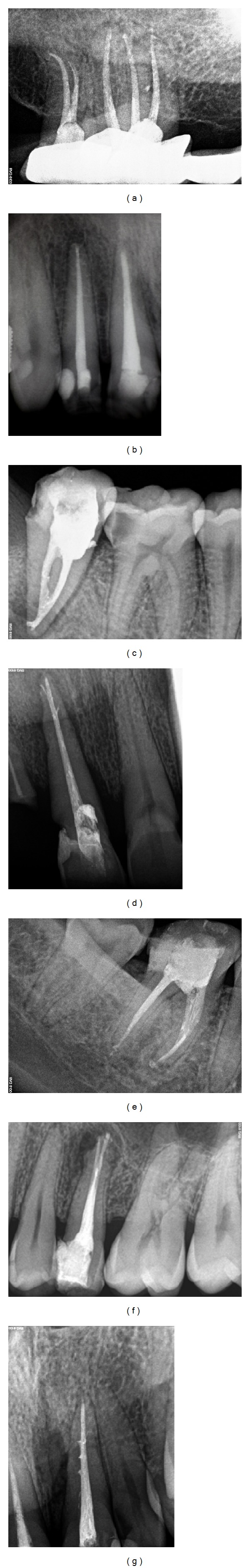
Clinical radiographs: (a) set 4, (b) set 3, (c) set 11, (d) set 10, (e) set 11, (f) set 6, and (g) set 1.

Based on the data presented in Figures 7 and 8, on the clinical observations we have done and with literature information, we can formulate a few observations regarding the shaping files in the ProTaper set. The impedance values of the three shaping files present a stable wear pattern. It is easily visible that the increase in resistance of these files in comparison to the finishing files is greater correlating to the clinical conditions of action. These files intervene in the first phase of canal enlargement, thus they are subjected to more wear. On the whole, S1 files present higher impedance values than S2 files. So, we can already get a grasp of the importance of flexibility and its effect on file mechanical properties. Also based on the Nyquist and Bode plots presented in Figure 4 correlated with Figure 5, we were able to theoretically assess the oxide layer modification per one use.

#### 3.3.2. Finishing Files

F1—It prepares the apical area of the canal. It is less elastic than the shaping files and has a more pronounced tendency of breakage as shown by West [3]. We can conclude based on our results that the reference value for these files was 37.8 Ohms and the highest value was of 113.4 Ohms, set number 2 (Figure 6). Usually the F1 files have a tendency to fracture more often being the first files to operate in the apical third conforming the apical preparation (Figures 3(a), 3(f), and 7).

F2—It enlarges the apical part of the canal and is less flexible than F1 described by West and Ruddle [3, 29]. There are studies that show interesting results by using only this one file in root canal enlargement, Yared [30], or that special filing technique could increase the instrument lifespan, De-Deus et al. [31]. The reference impedance value measured for this category of files was 37.4 Ohms and the highest value of the lot was in set number 2 registering 139.7 Ohms. The F2 and F3 are not used in all patients. The F2 and F3 can be too large and the enlargement to this file size could determine useless loss of tooth structure without any biostructural requirement. The counterargument would be that the hydraulics of correct root canal washing and filling may not be exercised correctly in an insufficiently tapered canal as shown by different authors, Fife et al., Ruddle, and Bukiet et al. [5, 29, 32]. This explains why there can be discrepancies regarding the wear of F2 and F3 files. Some sets may have a large number of uses but may have less worn F2 or F3 files and other less used sets may have more worn F2 and F3 files (Figure 7).

F3—It is the largest and less flexible file in the set described by West and Ruddle [3, 29]. It can only be used in straight canals because it has an apical transportation effect, Ruddle [29]. The reference impedance value for this file was 35.5 Ohms and the highest impedance value for F3 file is in set number 11 registering a value of 84.3 Ohms (Figure 7).

A few observations on the finishing files group can be formulated. The decrease of flexibility has a great impact on the mechanical properties of the files. This instance coupled with corrosion and mechanical stress leads to fracture. Although the finishing group has greater file diameters and should have a greater mechanical resistance, this feature proves to be a disadvantage when it comes to conforming curved dental canals. The wear pattern of finishing files is also apparent in Figures 7 and 8 presenting a lower increase in impedance values compared to the reference although surface defects were visible using the optical microscope (Figures 3(a), 3(d), and 3(f)).

Although file failure and intracanal separation could be catastrophic for the success of root canal therapy, we must bear in mind the difficult conditions in which rotary files act. They are constantly exposed to torsional stress in a corrosive environment by an operator with subjective sensitivity [3, 7, 10, 11]. Furthermore, improper handling in regard to disinfection and sterilization could cause further damage to files leading to failure [33]. EIS could prove to be a useful clinical tool for monitoring file clinical lifespan but much additional research is needed to understand its relationships to surface defects from the instrument manufacturing process and the crack-microstructure interactions that occur during fatigue failure under in vivo conditions.

## 4. Conclusions

Based on experimental EIS data for ProTaper we have seen that, with each clinical use, the oxide layer found on these instruments increases its impedance value. The change of oxide layer is in relation to the surface modifications to which the alloy is exposed during enlargement procedures, thus underlining EIS as a method to quantify the lifetime of each instrument. The fact that by comparing EIS data to optical microscope images we saw a direct relation to file degradation enables us to allege that our method could become a way to determine instrument clinical lifetime. EIS ohmic values that are double or triple the reference value were correlated through microscopy to surface alterations of the files. The novelty resides in a correlation between microscopy, clinical aspects, and EIS data providing a better understanding and registration of the ProTaper files clinical lifetime span even when used in curved canals.
